# WeChat-Based Intervention for Glycemic Control in Patients With Type 2 Diabetes Mellitus: Multicenter Randomized Controlled Trial

**DOI:** 10.2196/80738

**Published:** 2026-02-20

**Authors:** Chuanfen Zheng, Xiong Dou, Xiaotao Xiong, En-Yu Lei, Ming Jiang, Yulin Wu, Jing Yu, Xianjun Wang, Ling Zhang, Honghui Rong, Lu Lu, Fengju Li, Ting Luo, Xiangyu Ma, Ji-An Chen

**Affiliations:** 1 Department of Health Education, College of Preventive Medicine, Army Medical University, 30 Gaotanyan Street, Shapingba District, Chongqing, 400038, China, 86 02368771579; 2 Xinqiao Community Health Service Center, Shapingba District, Chongqing, China; 3 Chenjiaqiao Community Health Service Center, Shapingba District, Chongqing, China; 4 Yubeilu Community Health Service Center, Shapingba District, Chongqing, China; 5 Department of Epidemiology, College of Preventive Medicine, Army Medical University, Chongqing, China

**Keywords:** WeChat, glycemic control, type 2 diabetes, randomized controlled trial, WeChat mini program, health education tool

## Abstract

**Background:**

China’s diabetes epidemic faces critical gaps in glycemic control, with only 50.1% of treated patients achieving hemoglobin A_1C_ (HbA_1c_) targets in 2021. Conventional interventions struggle with scalability in primary care, particularly for vulnerable populations.

**Objective:**

This study aimed to evaluate the use of a WeChat-based health education tool (the WeWalk mini program, the Bayu Health public account, and a WeChat group) for improving glycemic control in community-dwelling patients with type 2 diabetes mellitus.

**Methods:**

This multicenter randomized controlled trial enrolled 600 adults with type 2 diabetes from 3 communities in Chongqing, randomly allocating participants 1:1 to either a 12-week WeChat-based intervention (n=300, 50%) or a control group (n=300, 50%) in September 2020. The control group received 4 face-to-face traditional health education sessions, whereas the intervention group participated in a digital program: a 4-week course followed by an 8-week practical implementation. At baseline and 12 weeks after the intervention began, both groups were examined in terms of HbA_1c_ and fasting blood glucose (FBG) as the primary outcomes, as well as variables such as blood lipid profile, blood pressure, and physical fitness–related indexes as secondary outcomes. Longitudinal glycemic control was assessed through triplicate FBG measurements extracted from standardized electronic health records at the 2-year follow-up. Independent *t* tests or Mann-Whitney *U* tests were used to assess changes from baseline to follow-up between groups.

**Results:**

A total of 92.7% (556/600) of the participants completed the 12-week follow-up visit. The WeChat-based intervention demonstrated superior glycemic control outcomes, with intervention participants achieving a 0.59% greater HbA_1c_ reduction than controls (−0.03% vs 0.56%; *P*<.001) and significant improvements in FBG levels (−0.69 vs 0.00 mmol/L; Δ=0.69; *P*=.001). Subgroup analysis revealed that WeChat-based health education was significantly effective in patients with diabetes with a disease duration of <10 years, educational level of junior high school or lower, and annual family income of <CN ¥50,000 (US $7172.10) . These benefits persisted throughout the 2-year follow-up, where the intervention group maintained lower FBG levels (6.87 vs 7.35 mmol/L; *P*=.001).

**Conclusions:**

WeChat-based health education was beneficial for glycemic control in primary health care settings. However, the sustained efficacy and feasibility of this approach require further investigation.

## Introduction

In recent years, the prevention and control of diabetes mellitus have become a global public health challenge, especially in China [1]. Effective management of type 2 diabetes mellitus (T2DM) relies critically on successful patient self-management [2], with structured diabetes self-management education (DSME) serving as a cornerstone of this process [3,4]. Despite this recognition, only 32.9% of patients with T2DM in China receive treatment, and among those treated, only 50.1% achieve adequate glycemic control [5]. This situation imposes substantial financial burdens on both patients and community health care systems [6,7].

Robust evidence from high-income countries, including Sweden [8], Spain [9], the United Kingdom [10], and New Zealand [11], has consistently demonstrated the efficacy of structured DSME programs in improving patient outcomes within primary care settings. These programs often emphasize lifestyle modifications, including balanced nutrition, regular physical activity, and weight management, as advocated by the World Health Organization [12]. Nevertheless, the traditional DSME model, which relies heavily on face-to-face and resource-intensive sessions, faces significant scalability challenges within the vast and heterogeneous primary health care environment [13]. Barriers to adopting these behaviors persist, including limited access to personalized health education and fragmented communication between patients and health care providers [14]. Therefore, building effective intervention systems for diabetes health education has become an important issue in primary health care in China.

The rapid proliferation of mobile health technologies has ushered in new opportunities for scalable diabetes management. Mobile apps have emerged as a cost-effective and efficient means of managing diabetes, primarily focusing on self-management and hospital appointments [15-17]. However, these apps often lack broad applicability for patients and family physicians in primary health care settings, neglecting the holistic needs of the community [18]. In contrast, ubiquitous social media platforms such as WeChat present a unique opportunity [19,20]. Their high penetration rate and familiar interface facilitate the delivery of structured educational content and enable continuous, interactive communication between patients and health care providers directly within the primary care context. Therefore, we designed an online free health education platform incorporating a WeChat mini program named WeWalk and a WeChat public account named Bayu Health.

This randomized controlled trial aimed to evaluate the effectiveness of WeChat-based interventions among patients with T2DM in Chinese community health centers. Our hypothesis was that mobile health education, which is based on the health belief model and social cognitive theory, would be more effective than conventional health education. We expected it to improve communication with community family physicians; foster peer-supportive social networks; promote healthy lifestyle changes; and ultimately, improve the effectiveness of diabetes self-management. By addressing these gaps and leveraging the potential of mobile health technology, this study contributes to the global effort in diabetes management.

## Methods

### Design

This study was designed as a 12-week, nonblinded randomized controlled trial to examine the efficacy of a WeChat-based intervention in patients with T2DM between September 2020 and December 2020. These participants were recruited from 3 community health service centers and followed up on at 12 weeks and 2 years after the intervention. Family physicians followed up on patients with diabetes via telephone to introduce the trial and determine potential participants’ willingness to take part. These participants had established health records and had signed up for contracted family physician services at 3 community health service centers in Chongqing, China. Before the trial started, all the researchers involved were trained in uniform protocols, including trial procedures, questionnaires, and the use of the WeChat mini program and public account. A CONSORT (Consolidated Standards of Reporting Trials) checklist was completed to guide the trial’s design, analysis, and findings (Checklist 1). Before conducting the formal research, we carried out a feasibility study, as shown in Multimedia Appendix 1.

### Participants

On the basis of the inclusion and exclusion criteria, the family physician teams selected eligible participants for registration. The inclusion criteria for enrollment were as follows: (1) participants who were diagnosed with T2DM and met the 1999 World Health Organization diagnostic criteria; (2) receipt of the contracted family physician service within the validity period; (3) ability of the participants to undergo face-to-face interviews and voluntary participation in the trial; and (4) ownership of a smartphone and familiarity with the WeChat app, including chatting, subscribing to WeChat public accounts, and using WeChat mini programs. The exclusion criteria for this study were as follows: (1) participants with severe complications, such as ketoacidosis or hyperosmolar nonketotic diabetic coma; (2) participants with severe heart disease who would not tolerate moderate-intensity exercise; (3) participants who could not answer the questions clearly; and (4) participants who were taking part in other intervention studies.

A total of 900 eligible patients with T2DM were recruited by family physician teams from 3 communities, of whom 300 (33.3%) were excluded. Of these 300 excluded patients, 196 (65.3%) did not meet the inclusion criteria, 79 (26.3%) refused to participate, and 25 (8.3%) were excluded for other reasons. A total of 600 participants were randomized into the intervention group (n=300, 50%) or the control group (n=300, 50%). Moreover, 556 participants completed the 12-week follow-up visit (intervention: n=278, 50%; control: n=278, 50%), and the retention rate was 92.7% (556/600).

### Sample Size

The sample size was calculated based on a completely random design using the tests for 2 proportions. The trial was designed for analysis via 2-tailed tests, with type I and II error rates set at 0.05 and 0.2, respectively. A priori sample size calculations were conducted for the primary outcome of hemoglobin A_1C_ (HbA_1c_) control rate (proportion of <7%). On the basis of national epidemiological data [1], the retention rate in the control group was estimated to be 35.2% in this study. We hypothesized that an 11% absolute risk reduction in the intervention group (24.2% event rate) derived from conservative adaptation of the Finnish Diabetes Prevention Study [21] would account for our digital intervention modality. With these parameters in the PASS software (version 15.0; NCSS, LLC), the total sample size required was 267 in each group. Considering a dropout rate of up to 10%, the final sample size was determined to be 300 patients in each group.

### Allocation

After the willingness of patients to participate in the study was confirmed by the family physician teams, the baseline data of the participants were collected at the community health service centers. Randomization was performed by an independent statistician who generated the allocation sequence through computerized random number generation. The participants were assigned in a 1:1 ratio to the study groups, with allocation concealment ensured via opaque, sealed envelopes that were sequentially numbered.

### Diabetes Health Education Team

Our multidisciplinary diabetes health education teams [22] were composed of the following: (1) a total of 18 family physicians from 6 family physician teams at 3 community health centers who were responsible for directly managing patients with diabetes and providing sustainable health education services; (2) an online health assistant who was responsible for functional maintenance and training on the health education platform; and (3) health education experts from the Military Health Education Center, including endocrinologists, rehabilitation therapists, ophthalmologists, dietitians, and coaches.

### WeChat-Based Health Education Tool

The WeChat-based health education tool used in our study consisted of the WeWalk mini program, the Bayu Health public account, and the WeChat group. The WeWalk mini program, an interactive mobile health platform, integrates 5 core functional modules designed to promote diabetes self-management through physical activity monitoring and community engagement. This gamified intervention system uses behavioral economics principles and social cognitive theory to enhance users’ health literacy and sustainable behavior change. Participants in the WeChat-based intervention group carried out daily health education activities to promote healthy behaviors. The WeChat public account Bayu Health was used to push diabetes self-management courses for the intervention group. The WeChat group was used mainly to organize and guide patients to participate in intervention activities. Multimedia Appendix 2 presents the functional modules of the WeWalk mini program and shows the QR codes of the WeWalk mini program and the Bayu Health public account.

### Intervention Protocol

#### Control Group

The participants in the control group received standard health education, consisting of 4 sessions over a 12-week period provided by their family physician team. The contents of the education intervention focused on 5 key areas: diabetes knowledge, dietary control, exercise therapy, drug therapy, and self-monitoring of blood glucose. Education was provided during regular monthly follow-up visits or scheduled clinic appointments through in-person consultations.

#### Intervention Group

Participants in the intervention group received a 12-week theory-based diabetes self-management program delivered through an integrated WeChat platform. The intervention was structured in 2 sequential, theory-informed phases to facilitate knowledge acquisition and sustainable behavior change.

##### Stage 1: Structured Education (Weeks 1‐4)

During the initial 4-week phase, the participants completed a structured self-management curriculum via the Bayu Health public account. Developed by the Military Health Education Center and aligned with China’s national guidelines, the curriculum incorporated the health belief model to increase perceived susceptibility, severity, benefits, and self-efficacy. It consists of 30 evidence-based microlearning modules (videos and articles) covering lifestyle modification, glycemic control, medication adherence, and complication prevention. Health assistants reinforced engagement through daily performance summaries and the recognition of active participants in dedicated WeChat groups.

##### Stage 2: Guided Application (Weeks 5‐12)

The subsequent 8-week phase focused on translating knowledge into practice via the WeWalk mini program. Grounded in principles of gamification and social cognitive theory, this stage included physician-supervised goal setting (Multimedia Appendix 3), photo-based tracking of physical activity and dietary patterns, and a gamified ranking system with virtual health points to incentivize consistent engagement. All user-generated health data were shared exclusively with the family physician teams via encrypted channels, enabling real-time clinical oversight while maintaining privacy.

### Data Collection and Measurements

The participants underwent 2 follow-up assessments: after the intervention (12 weeks) and in the long term (2 years after the intervention). To assess the condition of the patients, the primary outcome measure of the study was the change in fasting blood glucose (FBG) and HbA_1c_ levels in the 2 groups at baseline and 12 weeks after the intervention began. The secondary outcomes included changes in weight, BMI, waist circumference, hip circumference, systolic blood pressure, diastolic blood pressure, and serum lipid profiles. Baseline assessments included standardized questionnaires capturing demographics, medical and treatment history, comorbidities, and lifestyle factors, alongside fasting-state physical examinations conducted by certified personnel at community health service centers. Longitudinal glycemic control was assessed through triplicate FBG measurements extracted from standardized electronic health records at the 2-year follow-up.

### Statistical Analysis

Data were double entered using EpiData (EpiData Association) and analyzed using SPSS (version 26; IBM Corp). Normally distributed variables expressed as means and SDs were compared using independent *t* tests; nonparametric variables reported as medians and IQRs were analyzed via Mann-Whitney *U* tests. Demographic (sex, educational level, and annual family income), clinical (hypoglycemic medication use and diabetic complications), and behavioral (smoking and alcohol use) variables were analyzed using the chi-square test at baseline. The chi-square test was used to test the differences in HbA_1c_ data before and after the intervention. Patients with diabetes were divided into 2 categories according to their HbA_1c_ levels: HbA_1c_<7% (normal) and HbA_1c_≥7% (abnormal) according to the guidelines for the prevention and treatment of T2DM in China (2017) [23]. *P*<.05 was considered statistically significant.

### Ethical Considerations

This study was approved by the Army Medical University medical ethics committee (approval 2020-007-02) and registered at the Chinese Clinical Trial Registry (ChiCTR2300071926). Written informed consent was obtained from all participants before enrollment. All data were deidentified for analysis and stored securely to ensure confidentiality. Participants received no financial compensation but were provided with free medical checkups, medication guidance, and healthy lifestyle guidance.

## Results

**Figure 1.**
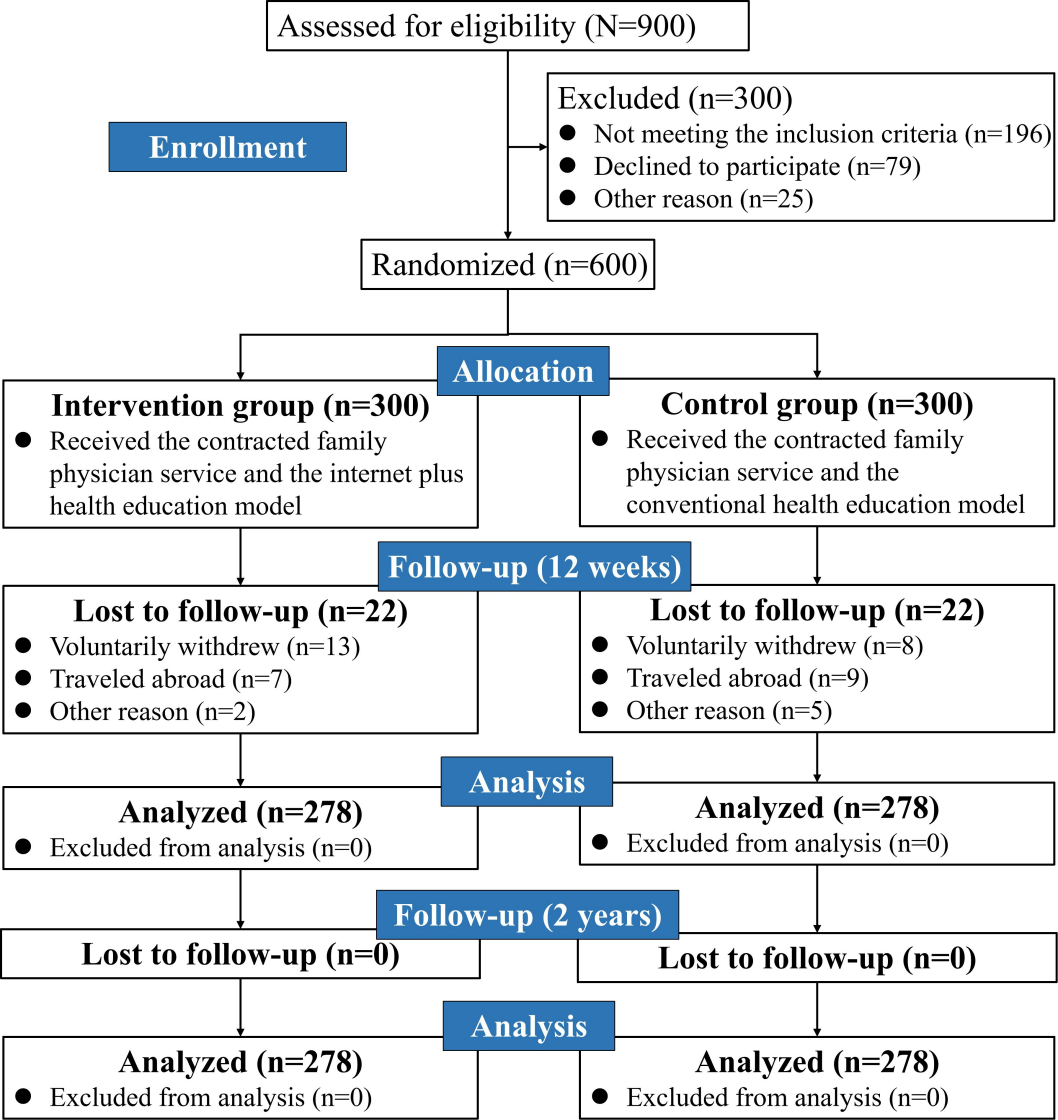
Participant flow diagram.

A total of 600 participants were randomized into the intervention or control group. Follow-up at 12 weeks was completed by 46.3% (n=278) of the participants per group (overall retention: n=556, 92.7%), with no further attrition at 2 years (Figure 1). The intervention and control groups were well balanced at baseline in terms of key demographic and clinical characteristics (Table 1).

**Table 1.** Baseline characteristics of the 2 groups. The chi-square test was used to compare 2 rates or multiple component ratios.

Characteristic	Intervention (n=278)	Control (n=278)	*P* value^a^
Age (years), mean (SD)	59.38 (6.93)	58.81 (6.21)	.312
Sex (female), n (%)	159 (57.2)	158 (56.8)	.932
Disease duration (years), mean (SD)	6.29 (4.50)	6.87 (5.29)	.166
**Educational level, n (%)**			.472
Primary school or lower	72 (25.9)	59 (21.2)	
Junior high school	118 (42.4)	131 (47.1)	
Senior high school	70 (25.2)	69 (24.8)	
College or higher	18 (6.5)	19 (6.8)	
**Annual family income, n (%)**			.144
<¥30,000 (US $4303.26)	64 (23.0)	61 (21.9)	
¥30,000-¥50,000 (US $4303.26-$7172.10)	96 (34.5)	91 (32.7)	
¥50,000-¥100,000 (US $7172.10-$14,344.20)	92 (33.1)	82 (29.5)	
>¥100,000 (US $14,344.20)	26 (9.4)	44 (15.8)	
Use of hypoglycemic drugs, n (%)	243 (87.4)	237 (85.3)	.459
Diabetic complications, n (%)	92 (33.1)	99 (35.6)	.532
**Smoking, n (%)**			.849
No	206 (74.1)	203 (73.0)	
Yes	51 (18.3)	57 (20.5)	
Quit	21 (7.6)	18 (6.5)	
**Alcohol consumption, n (%)**			.639
No	212 (76.3)	207 (74.5)	
Yes	55 (19.8)	60 (21.6)	
Quit	11 (4.0)	11 (4.0)	
BMI (kg/m^2^), mean (SD)	24.79 (3.05)	24.77 (3.44)	.941
Waist circumference (cm), mean (SD)	85.82 (9.02)	86.53 (8.83)	.352
Hip circumference (cm), mean (SD)	93.80 (7.02)	93.87 (7.00)	.902
WHR^b^, mean (SD)	0.92 (0.07)	0.92 (0.07)	.245
FBG^c^ (mmol/L), mean (SD)	8.12 (2.57)	8.39 (2.73)	.232
HbA_1c_^d^ (%), mean (SD)	7.39 (1.53)	7.44 (1.46)	.697
TC^e^ (mmol/L), mean (SD)	4.77 (1.14)	4.78 (1.09)	.954
TG^f^ (mmol/L), median (IQR)	1.70 (1.20-2.55)	1.91 (1.21-2.80)	.090
LDL-C^g^ (mmol/L), mean (SD)	2.55 (0.87)	2.50 (0.86)	.493
HDL-C^h^ (mmol/L), mean (SD)	1.31 (0.35)	1.30 (0.34)	.650
SBP^i^ (mm Hg), mean (SD)	129.63 (17.80)	128.67 (16.38)	.512
DBP^j^ (mm Hg), mean (SD)	77.82 (9.55)	77.09 (9.41)	.362

a Between-group differences for normally distributed variables were assessed using an independent-sample *t* test, whereas those for nonnormally distributed variables were assessed using a Mann-Whitney *U* test.

b WHR: waist-to-hip ratio.

c FBG: fasting blood glucose.

d HbA_1c_: hemoglobin A_1C_.

e TC: total cholesterol.

f TG: triglycerides.

g LDL-C: low-density lipoprotein cholesterol.

h HDL-C: high-density lipoprotein cholesterol.

i SBP: systolic blood pressure.

j DBP: diastolic blood pressure.

Compared with conventional education, after the 12-week intervention, the WeChat-based program resulted in statistically and clinically significant improvements in several metabolic outcomes, as detailed in Table 2. Participants in the intervention group achieved significantly greater reductions in FBG and HbA_1c_ at follow-up (*P*<.001 in both cases). A favorable effect was also observed on triglyceride levels, which were significantly lower in the intervention group (*P*=.003). The waist circumference and waist-to-hip ratio (WHR) levels in the control group were significantly higher than those in the intervention group. However, no significant between-group differences were found for total cholesterol, low-density lipoprotein cholesterol, high-density lipoprotein cholesterol, or blood pressure (*P*>.05 in all cases).

**Table 2.** The follow-up and change data of the 2 groups after 12 weeks of intervention.

Characteristic	Follow-up			Change		
	Intervention (n=278)	Control (n=278)	*P* value^a^	Intervention (n=278)	Control (n=278)	*P* value^a^
BMI (kg/m^2^), mean (SD)	25.37 (3.08)	25.53 (3.69)	.584	0.58 (1.81)	0.76 (1.81)	.245
Waist circumference (cm), mean (SD)	85.74 (8.35)	87.73 (8.78)	.006	–0.09 (5.85)	1.21 (6.70)	.016
Hip circumference (cm), mean (SD)	94.48 (7.23)	95.42 (7.82)	.143	0.68 (6.67)	1.55 (7.20)	.142
WHR^b^, mean (SD)	0.90 (0.06)	0.92 (0.06)	.019	–0.01 (0.07)	–0.00 (0.07)	.418
FBG^c^ (mmol/L), mean (SD)	7.43 (1.71)	8.39 (2.52)	<.001	–0.69 (2.42)	–0.00 (2.51)	.001
HbA_1c_^d^ (%), mean (SD)	7.35 (1.49)	8.00 (1.76)	<.001	–0.03 (1.83)	0.56 (1.60)	<.001
TC^e^ (mmol/L), mean (SD)	4.71 (1.02)	4.88 (1.07)	.059	–0.07 (1.05)	0.10 (0.96)	.058
TG^f^ (mmol/L), median (IQR) /mean (SD)	1.40 (1.03-2.00)	1.70 (1.10-2.69)	.003	–0.58 (1.88)	–0.25 (1.59)	.025
LDL-C^g^ (mmol/L), mean (SD)	2.60 (0.84)	2.59 (0.85)	.876	0.05 (0.72)	0.09 (0.75)	.529
HDL-C^h^ (mmol/L), mean (SD)	1.34 (0.36)	1.33 (0.33)	.558	0.03 (0.23)	0.03 (0.29)	.861
SBP^i^ (mm Hg), mean (SD)	133.62 (14.48)	134.23 (16.02)	.633	3.99 (16.46)	5.56 (17.25)	.273
DBP^j^ (mm Hg), mean (SD)	79.33 (8.67)	79.39 (9.92)	.940	1.51 (10.19)	2.31 (9.91)	.353

a Between-group differences for normally distributed variables were assessed using an independent-sample *t* test, whereas those for nonnormally distributed variables were assessed using a Mann-Whitney *U* test.

b WHR: waist-to-hip ratio.

c FBG: fasting blood glucose.

d HbA_1c_: hemoglobin A_1C_.

e TC: total cholesterol.

f TG: triglycerides.

g LDL-C: low-density lipoprotein cholesterol.

h HDL-C: high-density lipoprotein cholesterol.

i SBP: systolic blood pressure.

j DBP: diastolic blood pressure.

The rate of normal HbA_1c_ levels was not significantly different between the 2 groups at baseline (*P*=.932), whereas it decreased significantly in the control group after the intervention (*P*=.001), as shown in Table 3. Subgroup analyses were performed according to different characteristics (sex, disease duration, educational level, and annual family income), and detailed data are shown in Table 3. Significant differences in the rate of normal HbA_1c_ levels were observed between the 2 groups after the intervention in the participants with disease duration (<10 years), educational level (junior high school or lower), and annual family income [<CN ¥50,000 (US $7172.10)].

**Table 3.** Distribution of hemoglobin A_1C_ (HbA_1c_) levels before and after the intervention.

Characteristic	Baseline			12 week		
	HbA_1c_<7%, n/N (%)	HbA_1c_≥7%, n/N (%)	*P* value^a^	HbA_1c_<7%, n/N (%)	HbA_1c_≥7%, n/N (%)	*P* value^a^
**Overall**			.932			.001
Intervention	132/278 (47.5)	146/278 (52.5)		129/278 (46.4)	149/278 (53.6)	
Control	131/278 (47.1)	147/278 (52.9)		92/278 (33.1)	186/278 (66.9)	
**Sex**						
**Female**			.466			.033
Intervention	75/159 (47.2)	84/159 (52.8)		73/159 (45.9)	86/159 (54.1)	
Control	81/158 (51.3)	77/158 (48.7)		54/158 (34.2)	104/158 (65.8)	
**Male**			.333			.015
Intervention	57/119 (47.9)	62/119 (52.1)		56/119 (47.1)	63/119 (52.9)	
Control	50/120 (41.7)	70/120 (58.3)		38/120 (31.7)	82/120 (68.3)	
**Disease duration (years)**						
**<10**			.756			.001
Intervention	113/214 (52.8)	101/214 (47.2)		110/214 (51.4)	104/214 (48.6)	
Control	100/196 (51.0)	96/196 (49.0)		70/197 (35.5)	127/197 (64.5)	
** ≥10**			.353			.737
Intervention	19/64 (29.7)	45/64 (70.3)		19/64 (29.7)	45/64 (70.3)	
Control	30/81 (37.0)	51/81 (63.0)		22/81 (27.2)	59/81 (72.8)	
**Educational level**						
**Junior high school or lower**			.537			<.001
Intervention	90/190 (47.4)	100/190 (52.6)		90/190 (47.4)	100/190 (52.6)	
Control	84/190 (44.2)	106/190 (55.8)		53/190 (27.9)	137/190 (72.1)	
**Senior high school or higher**			.451			>.999
Intervention	42/88 (47.7)	46/88 (52.3)		39/88 (44.3)	49/88 (55.7)	
Control	47/88 (53.4)	41/88 (46.6)		39/88 (44.3)	49/88 (55.7)	
**Annual family income**						
**<CN ¥ 50,000 (US $7172.10)**			.908			<.001
Intervention	80/160 (50.0)	80/160 (50.0)		76/160 (47.5)	84/160 (52.5)	
Control	75/152 (49.3)	77/152 (50.7)		43/152 (28.3)	109/152 (71.7)	
**≥CN ¥ 50,000 (US $7172.10)**			.953			.340
Intervention	52/118 (44.1)	66/118 (55.9)		53/118 (44.9)	65/118 (55.1)	
Control	56/126 (44.4)	70/126 (55.6)		49/126 (38.9)	77/126 (61.1)	

a The chi-square test was used to compare 2 rates.

Two years after the intervention, the FBG level was significantly lower in the intervention group than in the control group (mean 6.87, SD 1.48 mmol/L vs mean 7.35, SD 1.87 mmol/L; *P*=.001), but there was no significant difference in the change in FBG from baseline to the 2-year follow-up between the 2 groups. Among the participants with a disease duration of <10 years, the FBG level was significantly lower in the intervention group than in the control group (mean 6.82, SD 1.43 mmol/L vs mean 7.42, SD 2.00 mmol/L; *P*=.001). Among the participants with annual family income of <CN ¥50,000 (US $7172.10), the FBG level was significantly lower in the intervention group than in the control group (mean 6.94, SD 1.56 mmol/L vs mean 7.53, SD 2.24 mmol/L; *P*=.007; Table 4).

**Table 4.** Follow-up fasting blood glucose data 2 years after the intervention.

Characteristic	Follow-up (2 years; mmol/L), mean (SD)			Change (2 years; mmol/L), mean (SD)		
	Intervention	Control	*P* value^a^	Intervention	Control	*P* value^a^
Overall	6.87 (1.48)	7.35 (1.87)	.001	−1.25 (2.87)	−1.04 (3.14)	.403
**Sex**						
Female	6.91 (1.67)	7.29 (2.00)	.062	−1.11 (2.76)	−0.93 (3.11)	.569
Male	6.82 (1.17)	7.42 (1.68)	.001	−1.44 (3.00)	−1.20 (3.19)	.534
**Disease duration (years)**						
<10	6.82 (1.43)	7.42 (2.00)	.001	−1.13 (2.73)	−0.71 (3.01)	.150
≥10	7.03(1.61)	7.17 (1.52)	.588	−1.67 (3.28)	−1.84 (3.31)	.757
**Educational level**						
Junior high school or lower	6.95 (1.64)	7.37 (1.91)	.024	−1.32 (3.03)	−1.10 (3.31)	.504
Senior high school or higher	6.68 (1.03)	7.31 (1.18)	.005	−1.12 (2.48)	−0.91 (2.75)	.606
**Annual family income**						
<CN ¥50,000 (US $7172.10)	6.94 (1.56)	7.53 (2.24)	.007	−0.87 (2.64)	−0.71 (3.28)	.624
≥CN ¥50,000 (US $7172.10)	6.77 (1.36)	7.13 (1.26)	.034	−1.77 (3.08)	−1.45 (2.92)	.394

a Between-group differences were assessed using the Mann-Whitney *U* test.

## Discussion

### Principal Findings

This study evaluated the short-term efficacy of a WeChat-based health education tool in primary care through a 12-week randomized trial with a 2-year follow-up on glycemic control. After 12 weeks, the intervention group had improved HbA_1c_ and FBG levels compared with those of the control group, especially in patients with diabetes with a disease duration of <10 years, an educational level of junior high school or lower, and a annual family income of <CN ¥50,000 (US $7172.10) . Significant between-group differences were found in waist circumference and triglycerides, but no significant changes were observed in BMI, hip circumference, WHR, total cholesterol, low-density lipoprotein cholesterol, high-density lipoprotein cholesterol, systolic blood pressure, or diastolic blood pressure. Two years after the intervention, FBG levels were significantly lower in the intervention group. However, the change in FBG from baseline was not significantly different between the groups.

### Comparison With Prior Work

Our study adds to the growing body of international evidence confirming the efficacy of digital health interventions, including smartphone apps and telemedicine, in improving diabetes outcomes [24-26]. This global consensus is strongly supported by a series of studies in China that have demonstrated the effectiveness of telemedicine and app-based interventions for glycemic control [27-29]. Specifically, with respect to the WeChat platform, our results are consistent with and reinforce findings from regional studies in the Jiangsu [19], Jilin [30], and Henan [31] provinces. The convergence of evidence from these diverse settings solidifies the view that WeChat-based health education is a viable and effective strategy for improving blood glucose levels in patients with T2DM. The intervention likely works by first improving disease knowledge through structured education [20]; then increasing self-efficacy via interactive support [32]; and, finally, enhancing adherence to medication and healthy behaviors through regular reminders and self-monitoring [33].

We found that the reduction in the HbA_1c_ level in the intervention group was lower than that reported in other studies according to a systematic review of self-management among patients with type 2 diabetes via the WeChat app [34]. A similar eHealth intervention in Shanghai demonstrated a greater HbA_1c_ reduction (7.9% to 7.3%) than our study (7.39% to 7.35%) [28]. This difference may be attributed to regional disparities in health care infrastructure [35-37] and a critical design variation: their single-platform (WeChat public account) intervention likely offered a simpler user experience, whereas our multimodule design incorporating a mini program may have increased operational complexity for participants.

We found that WeChat-based health education improved the HbA_1c_ level in patients with diabetes with low income, low educational level, and short disease duration who contracted family physicians. Currently, health apps designed to enhance self-management of chronic illnesses require continuous patient involvement as a pivotal factor influencing clinical effectiveness [38-40]. A meta-analysis revealed that the dropout rate in clinical trials for smartphone apps was significantly higher among high-income countries than among lower-middle–income countries [41]. A national web-based survey in China revealed that educational level and family income were associated with diabetes management app use in adult patients [42]. We speculated that patients with diabetes with shorter disease durations would have better acceptance of the WeChat-based health education tool, those with low educational levels would have higher expectations for our intervention, and our free program would be more attractive to low-income patients with diabetes.

Moreover, we found that the waist circumference and WHR in the intervention group were significantly lower than those in the control group. Indeed, sex was a confounding factor, with the waist circumference and WHR of female patients being out of balance in the 2 groups before the intervention. We observed that the BMI values increased in both groups after the intervention, presumably related to the end of the intervention in the winter. People are more likely to eat more food during the Spring Festival in China [43,44]. However, WeChat-based health education was effective in helping patients with diabetes control their body shape in the winter, which was similar to the findings of other studies [45].

The intervention was constructed based on the WeChat mini program and public account, which enhances communication between family physicians and patients [46]. The family physicians’ online virtual clinics were set up on the WeWalk mini program to help patients easily contact their contracted family physicians. Compared with other diabetes management mobile apps, the WeWalk mini program is free to download and use and easy to operate, reducing the likelihood of users discontinuing use [42]. Furthermore, subgroup analysis revealed that WeChat-based health education was effective for glucose control based on evidence from 3 communities. The WeChat mini program provides remote supervision and ongoing support for patients and physicians, which makes it easy to achieve continuous efficacy in the long term [47]. We suggest integrating effective, low-cost digital solutions such as this WeChat mini program into national chronic disease management strategies. Further validation is needed to assess its efficacy in rural settings with different health care infrastructures.

### Limitations

Our study has several limitations. First, due to the impact of COVID-19, formal follow-up was only conducted at 12 weeks, and only the short-term effects of WeChat-based health education were observed in our study. Regarding the long-term effects of WeChat-based health education, only FBG was evaluated in this study. However, this result still needs to be further evaluated through other indicators. Second, this study was based on 3 community health service centers but was not a cluster randomized controlled trial, which may lead to insufficient accuracy of the study results. Although the authorized supervisor prevented information contamination, they may have unintentionally disclosed intervention details while managing both groups. Third, no implementation fidelity data such as user engagement and use frequency of the intervention group were collected and analyzed. Finally, a future cost-effectiveness analysis and continuous real-world optimization are crucial next steps to definitively inform policy. We could explore incorporating artificial intelligence to provide more personalized, patient-specific educational content and support, thereby enhancing the scalability and integration of this WeChat-based intervention.

### Conclusions

We conducted a randomized controlled trial in community-dwelling patients with T2DM to examine the efficacy of a WeChat-based health education intervention in Chongqing, China. By strategically integrating social media platforms with primary care services, this WeChat-based health education intervention achieved significant results in improving glycemic control in patients with T2DM. Future investigations should use longitudinal designs to evaluate the sustained efficacy and feasibility of implementing WeChat-based health education.

## Supplementary material

10.2196/80738 Multimedia Appendix 1 Feasibility study.

10.2196/80738 Multimedia Appendix 2 The introduction of the WeChat mini program and public account.

10.2196/80738 Multimedia Appendix 3 Descriptions and screenshots of diabetes self-management goals organized into 16 items.

10.2196/80738 Checklist 1 CONSORT-eHEALTH (Consolidated Standards of Reporting Trials of Electronic and Mobile Health Applications and Online Telehealth) checklist (V 1.6.1).

## Abbreviations

CONSORT: Consolidated Standards of Reporting Trials
CONSORT-EHEALTH: Consolidated Standards of Reporting Trials of Electronic and Mobile Health Applications and Online Telehealth
DSME: diabetes self-management education
FBG: fasting blood glucose
HbA_1c_: hemoglobin A_1C_
T2DM: type 2 diabetes mellitus
WHR: waist-to-hip ratio
