# Supplementary material for: WeChat-Based Intervention for Glycemic Control in Patients With Type 2 Diabetes Mellitus: Multicenter Randomized Controlled Trial
Source: JMIR Mhealth Uhealth. 2026 Feb 20;14:e80738. doi: 10.2196/80738 (PMC12923094; doi:10.2196/80738)
Supplement: Multimedia Appendix 1 [file mhealth-v14-e80738-s001.pdf]

Multimedia Appendix 1 Feasibility study.

From April to June 2020, seven community volunteers with a mean age of 32 years were invited to participate in the feasibility pre-experiment of the WeChat mini program and public account. The researchers informed the volunteers of the purpose of testing the WeChat mini program and public account. After obtaining their consent, all volunteers were trained to use the network platform to ensure that they completed each module under the guidance of the family physician team. Volunteers stated that the operation interfaces of the WeChat mini program and public account were simple and easy to use. It was convenient for them to communicate with family physicians, participate in online health education activities, and record daily lifestyle. Additionally, the video played smoothly on the WeChat public account.
