# Supplementary material for: WeChat-Based Intervention for Glycemic Control in Patients With Type 2 Diabetes Mellitus: Multicenter Randomized Controlled Trial
Source: JMIR Mhealth Uhealth. 2026 Feb 20;14:e80738. doi: 10.2196/80738 (PMC12923094; doi:10.2196/80738)
Supplement: Multimedia Appendix 2 [file mhealth-v14-e80738-s002.pdf]

Multimedia Appendix 2 The introduction of the WeChat mini program and public account.

The WeWalk mini program was a WeChat-based mobile health platform integrating step-counting technology with gamified behavioral activation to enhance diabetes self-management, which was consisted of five core modules.

1. Adaptive Step Monitoring System: Utilizing WHO physical activity guidelines, the system categorizes daily steps via tri-color coding: <6,000 (yellow/inactive), 6,000-20,000 (green/target), >20,000 (red/excessive). Threshold-triggered alerts provide personalized exercise prescriptions compliant with American Diabetes Association recommendations.

2. National Health Lifestyle Module: Family physicians deliver government-supported interventions through virtual step competitions and evidence-based nutrition seminars aligned with China's Dietary Guidelines. Real-time leaderboards and moderated discussion forums enhance health literacy dissemination.

3. Health Circle Module: It includes health activities circle and family physicians circle. The health activity circle is used to invite users to participate in various health activities, record the users' exercise and diet, join the topic discussion group, and enhance the interactive support among users. The family physicians circle is the online studio of family physician teams in which family physicians can pre-sign with users, manage the lifestyle of the users, and provide with the point-to-point consultation.

4. Gamified Incentive Mechanism: A token economy rewards step milestones and

educational task completion via virtual health points. Accumulated points redeem diabetes-specific utilities, applying operant conditioning principles to reinforce health behaviors.

5. Health Analytics Dashboard: Record the user’s basic health status, virtual health points, behavioral trends (weekly step variance), and social engagement metrics. Keep track of the posts and comments from user's health circle.

The home page and functional modules are shown in Appendix figure 1, and the quick response (QR) codes of the WeWalk mini program and Bayu Health public account are shown in Appendix figure 2.

Appendix figure 1. The homepage and functional modules of the WeWalk mini program.

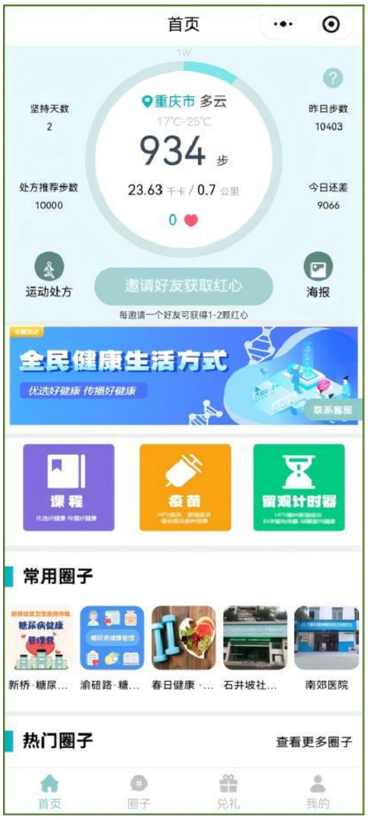

Homepage

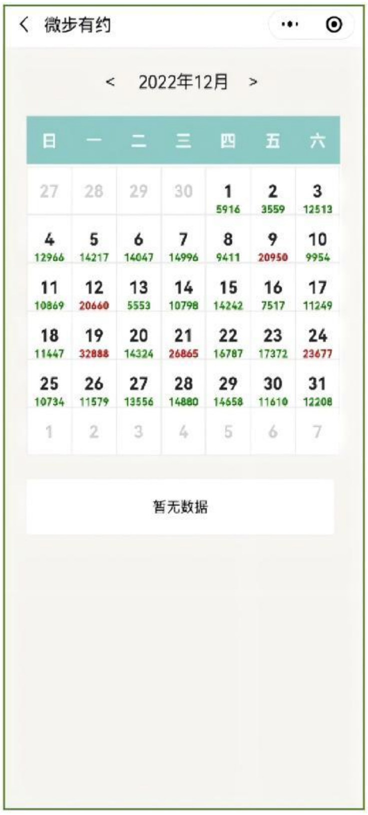

Module 1

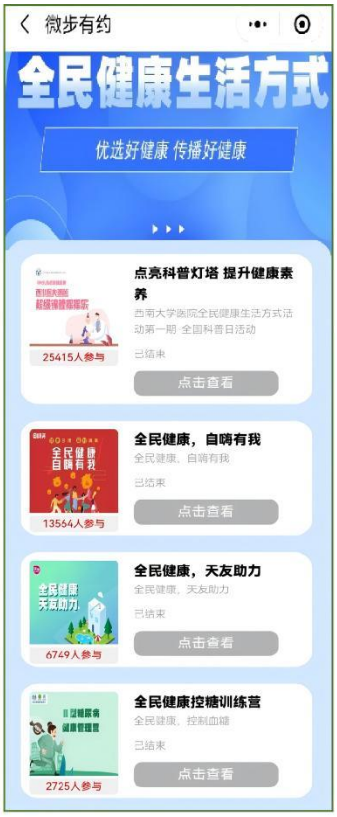

Module 2

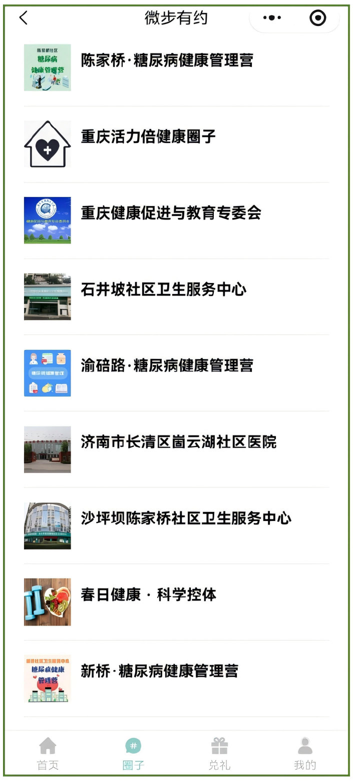

Module 3

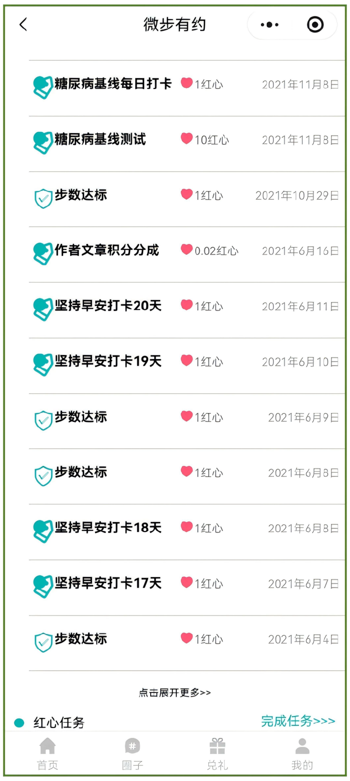

Module 4

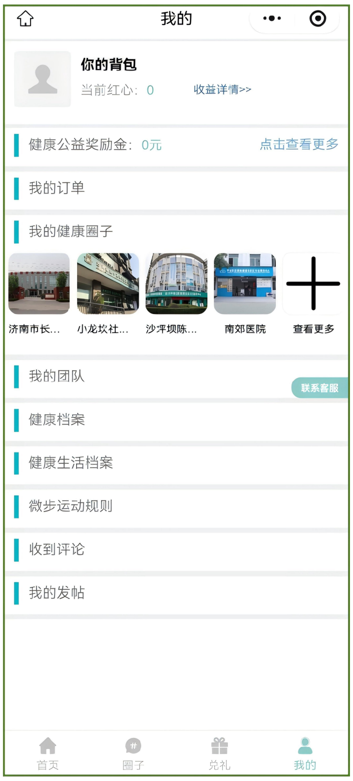

Module 5

Appendix figure 2. The quick response (QR) codes of the WeWalk mini program and Bayu Health public account.

The QR code for
"WeWalk" mini program

The QR code for
"Bayu Health" public account
