# Supplementary material for: WeChat-Based Intervention for Glycemic Control in Patients With Type 2 Diabetes Mellitus: Multicenter Randomized Controlled Trial
Source: JMIR Mhealth Uhealth. 2026 Feb 20;14:e80738. doi: 10.2196/80738 (PMC12923094; doi:10.2196/80738)

Multimedia Appendix 3 Descriptions and screenshots of diabetes self-management goals organized into 16 items.

No.	Items
1	Monitor blood glucose
2	Active follow-up
3	Exercise plan
4	Self-management
5	Blood glucose level
6	Food choice
7	Regular follow-up
8	Take medicine as prescribed
9	Record blood glucose
10	Regular exercise
11	Strict diet
12	Dessert intake
13	Proactive medical care
14	Physical exercise
15	Timed medication
16	Eat and drink unreasonably
Note: Red indicator represented the issue requiring attention, indicating that the behavior item was not well managed, and the patient needed to improve this behavior. Green indicator represented satisfactory performance, indicating that the behavior item was well managed.	

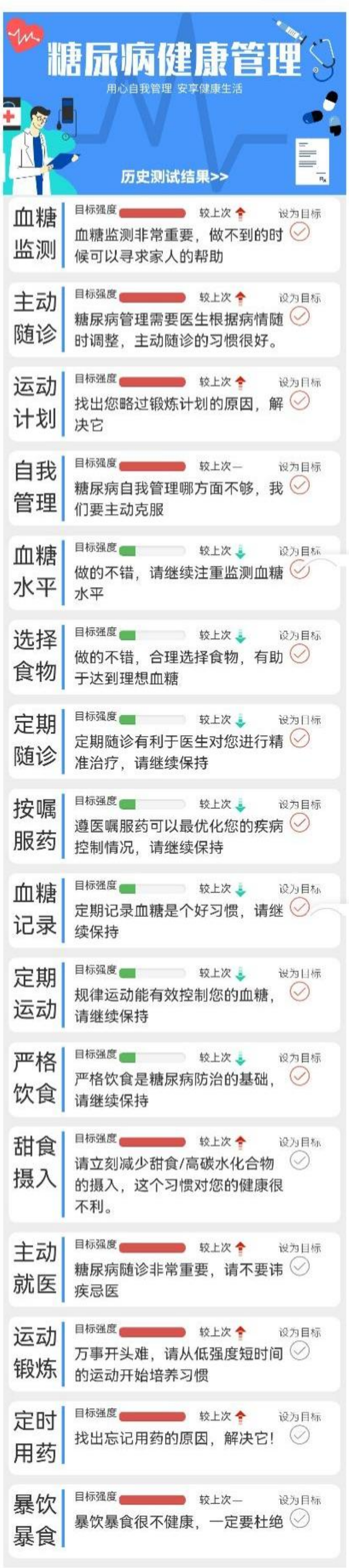

Supplement: Multimedia Appendix 3 [file mhealth-v14-e80738-s003.pdf]
